# Prevention of Diseases in the War

**Published:** 1918-11

**Authors:** 


### Prevention of Diseases in the War.

The American Defense Society is making an earnest effort for a proper reorganization of the Medical Department of the Army, and in sending out a leaflet written by Dr. Louis Livingston Seaman, late Surgeon Major, U. S. Volunteer Engineers, asks all who are in sympathy with the effort to write and request congress to pass the measure now pending which provides for Medical Department reorganization.

Dr. Seaman says:

"The Bill now pending before Congress for the reorganization of the Medical Department of the Army is of as grave importance as any measure that has been presented since the American nation entered the present war, and its fate *may* determine the final issue of the war. When it is remembered that the Medical Department has to combat a foe, that in all the great wars of history, excepting the Russo-Japanese, has caused 80 per cent. of the entire mortality—never less than four times, and often twenty times as many as the artillery, infantry, shells and all other methods of physical destruction combined, the responsibility and importance of the medical officer in war will be appreciated.

"The Department he represents has never had the necessary authority to enable it to reduce this frightful eighty per cent mortality to a minimum, and to do so without in any way interfering with the strategy, or military operations of the war.

"The Medical Department of our Army is founded on the traditions of the British Medical Department of 1776, when preventive medicine was an unknown science, and the duty of the medical officer was to cure disease, instead of preventing it —of locking the stable door after the theft had been committed.

"Our medical officers have never had the necessary rank and authority to prevent the development of the epidemics and other diseases in our Army that have caused the frightful mortality incident to War. Witness the records of the Spanish-American War in Cuba and Porto Rico and in the Philippines, which practically typify the conditions that existed in the Boer War in South Africa, in our own Civil War of 61-65, in the Russo-Turkish War, and in the British campaign in the Crimea.

"The Porto-Rican Expedition in the opera bouffe performance known as the Spanish War may be taken as an example, for nowhere in history is there found a more illuminating instance, a graver lesson, or a more terrible warning than is there portrayed. For our country, it is the 'Mene, Mene, Tekel Upharsin,'—the handwriting on the wall, so easily decipherable that he who runs may read; and yet, in the glory of victory, and the enjoyment of prosperity, its lesson has passed unheeded.

"The story of the Expedition is brief. About 20,000 American troops landed in Porto Rico, while the Spanish on the Island numbered about 17,000. Several skirmishes occured, in which, *according to the Surgeon General's report,* three men were lost from the casualties of war. The object of the war, the breaking of the chains of Spanish despotism and spoliation, which for centuries had held a race in shameful moral serfdom, was soon accomplished, and the war—from the strictly military standpoint, was over. From our first arrival, the natives of the island welcomed our battalions with vivas of applause, strewing our advancing march with flowers, and their masses were prepared to joyfully second our efforts for their complete emancipation.

"That is the beautiful story history presents. Lest we forget, as a Nation, and lie supine in the easy content of this picture, let me invite attention for a moment to a further study of the report of the Surgeon General for that war. It states that, although only three men fell from the casualities of battle during that entire campaign in Porto Rico, 262, or nearly one hundred times as many, died from preventable causes. It fails, however, to state that the number of hospital admissions nearly equaled the entire strength of the invading army, and that the camps of the army, from one end of the island to the other, were pestiferous hot-beds of disease, before they had been occupied a month; so that, had the bugle sounded for action, only a small percentage of the units would have been in a condition to respond to the call. Nor was this state of affairs confined to Porto Rico. In the invading armies of the Philippines and Cuba the same conditions prevailed.

"The official figures as shown on the following table were furnished me *by the Surgeon General of the Army,* on the 10th day of October, 1905, and cover the vital statistics of the United States Military Expeditions for the year 1898.

| | Deaths from Battle Casualties. | Deaths from Disease. |
|---|---|---|
| In the Philippine Islands | 17 | 203 |
| In Porto Rico | 3 | 262 |
| In Cnba | 273 | 567 |
| In the U. S. Home Camps, etc. | | 2,649 |
| Total deaths | 293 | 3,681 |

Or about one from casualties to thirteen from disease.

"The report further shows that while the average mean strength of the army enlisted for the Spanish War was about 170,000, the total number of admissions to the hospitals was on September 10, 1898, over 158,000, or *90 per cent.* This in a war of less than three months duration, and in which more than three-fourths of its soldiers never left the camps of their native land.

"The Japanese army for the same period had about 4 per cent. hospital admissions, or one twenty-second times as many.

"The vast difference in favor of the Japanese figures illustrates the value of a medical and sanitary department properly equipped to enforce practical sanitation, dietary, and other preventive measures.

"The greatest tragedy of War lies not on the battle field but in the failure of a government to protect its guardians from *preventable diseases,* thereby immeasurably increasing the suffering and mortality incident to it. This can be largely prevented by giving the medical officer authority to enforce sanitation, and supervisory control over the rations of the troops.

"Every death from preventable disease is an insult to the intelligence of the age. If it occurs in the army, it becomes a governmental crime. From the beginning the State deprives the soldier of his liberty, prescribing his hours of rest, his exercise, equipment, dress, diet, and the locality in which he shall reside; and in the hour of danger it expects him, if necessary, to lay down his life in defence of its honor. It should, therefore, give him the best sanitation and the best medical supervision the science of the age can devise, be it American, Japanese or Patagonion,—a fact of which Congress will do well to take cognizance at the earliest moment. For, just as surely as the engineer who disregards the signals, or the train dispatcher who gives wrong orders, is legally responsible for the loss of human life in the wreck which follows, so Congress, or the medical system of our Army, is responsible for all soldiers' lives that are needlessly and criminally sacrificed,—not on the glorious field of battle, but in diseased camps, from preventable causes.

"Herbert Spencer, in his 'Synthetic Philosophy' refers to 'the ill treatment accorded the medical officers of the English Army as a late survival of the days of feudalism, and contempt for the purely scientific.'

"If wars are inevitable, and the slaughter of men must go on

(and I believe wars are inevitable, and that most of them are ultimately beneficial), then let our men be killed legitimately on the field, fighting for the stake at issue, and not dropped by the wayside from preventable disease, as they did in the Spanish-American War—1,300 for every 100 that died in action. It is for the 1,300 brave fellows who are needlessly sacrificed, *never* for the 100 who fall gallantly fighting, that I offer my prayer.

"I believe that if our Medical Department in the Spanish-American War had been systematized, with sufficient numbers, *with supervisory control over the ration, and with power to enforce sanitary and hygienic regulations,* the men of our army would have returned to their homes at the close of the campaign in better physical condition than when they entered it, improved by their summer outing.

"An army might be suffering from diarrhea or slight intestinal catarrh, due to change of water, of ration, or climate (and I have seen 90 per cent. of an entire command in this condition at one time), compelled to live on a diet of pork and beans and fermented canned rubbish that in six weeks prostrated 50 per cent. of its number with intestinal diseases, and sent three thousand to their everlasting homes, to say nothing of the enormous number invalided, and the seventy-five thousand pension claims that followed as the result. Until the men were admitted to hospital wards the medical officer had no authority to even order a rice diet, which would have prevented the men from becoming invalids. *This was one of the principal causes that brought our army of 170,000 men in the Spanish War almost to its knees in three months, and sent the survivors home in the shrunken and shriveled condition which many of us still remember.*

"In all the wars in which the United States have engaged, disease has been responsible for more than 70 per cent. of the mortality, more than half of which could have been easily prevented had the Medical Department been properly empowered to meet its obligations. *Preventable disease, more than wounds, swells the pension list.* Statistics of the Pension Office prove that if this unnecessary loss had been avoided, the saving in pensions alone, in every war in which America has participated, would have paid the cost of the resulting war in every twenty-five years. Aside from the sorrow of the homes made desolate, consider the economic value of the 70 per cent. of lives needlessly sacrificed, that might have been saved as breadwinners in industrial pursuits.

"In an address delivered before the International Congress of Military Surgeons in 1904, after my return from the Russo-Japanese War, I said:

" 'Perhaps the day is not distant when another summons will come to join the Army of the Republic, when the first call may be, not as in the Civil War for 75,000 men, nor as in the Spanish War for 250,000, but when, more likely it will be for a round half million, to be followed, possibly by another of equal number. And the question will be asked by the young patriot of that day, not '*who* is the enemy to be met,'—no, the American boy is not built that way,—but he will demand to know *what measures have been taken to insure him against the silent enemy that kills the eighty per cent.* And when he learns the same prehistoric regulations as to sanitation and protection against this foe are in force as existed in 1904, will he respond to his country's call? Yes he will—for that is the way the American boy *is* built. And he will follow, as did his forbears, in their footsteps; and he will fall by the wayside as they did before. And history will record another crime.

" 'We see by the light of thousands of years,
And the knowledge of millions of men,
The lessons they learned through blood and in tears
Are ours for the reading, and then
We sneer at their errors and follies and dreams,
Their frail idols of mind and of stone,
And call ourselves wiser, forgetting it seems,
That the future may laugh at our own.'

Give the Medical Officer rank, and *authority,* in all matters appertaining to sanitation and preventable disease, and supervision over the ration, when such authority will not interfere with the strategy of the officer of the line; and then, if epidemics or other preventable diseases occur, have him court-martialed and cashiered from the Army, as though he were a traitor and a spy."

---

His Warm Reception.—Doctor: "You mustn't stay out late at night."

Patient (a married man): "Is the night air bad for me?"

Doctor: "No, it's the excitement after getting home that hurts you."

